# Editorial: Explainable and advanced intelligent processing in the brain-machine interaction

**DOI:** 10.3389/fnhum.2023.1280281

**Published:** 2023-09-12

**Authors:** Xiaofeng Xie, Dingguo Zhang, Tianyou Yu, Yucong Duan, Ian Daly, Shenghong He

**Affiliations:** ^1^Mechanical and Electrical Engineering College, Hainan University, Hainan, Haikou, China; ^2^Centre for Autonomous Robotics (CENTAUR), Department of Electronic and Electrical Engineering, University of Bath, Bath, United Kingdom; ^3^School of Automation Science and Engineering, South China University of Technology, Guangzhou, China; ^4^Pazhou Lab, Guangzhou, China; ^5^Department of Data Science and Big Data Technology, Hainan University, Haikou, China; ^6^Brain-Computer Interfaces and Neural Engineering Laboratory, School of Computer Science and Electronic Engineering, University of Essex, Essex, United Kingdom; ^7^MRC Brain Network Dynamics Unit, Nuffield Department of Clinical Neurosciences, University of Oxford, Oxford, United Kingdom

**Keywords:** interpretability, brain machine interface, brain computer interface, machine learning, EEG, fNIRS, deep brain stimulation

Brain-machine interfaces (BMIs), also known as brain-computer interfaces (BCIs), allow their users to control external devices directly using brain signals without relying on the peripheral nervous system and muscles (Wolpaw et al., 2002). As a new bioengineering technology, BCI has a great potential in motor function enhancement to help patients disabled by diseases, such as stroke. In particular, BCI systems with interactive brain stimulation, such as deep brain stimulation (DBS), provide an active way to reveal relationships underlying the interplay between body control and brain activities, as well as to better understand pathological mechanisms underlying diseases such as Parkinson's disease (PD) and essential tremor (ET). While developing such advanced BCI systems, one of the major challenges is to apply explainable and advanced intelligent processes to decode the information embedded in brain signals such as EEG and functional Near-Infrared Spectroscopy (fNIRS) recorded non-invasively from scalp, or local field potential (LFP) recorded invasively from cortical (e.g., motor cortex) or subcortical (e.g., thalamus) brain structures (Heldman et al., 2006; Opri et al., 2020; He et al., 2021).

The goal of this Research Topic for the Brain-Computer Interface section of *Frontiers in Human Neuroscience* is to collect the current developments of explainable and advanced intelligent methods in BCIs based on EEG, fNIRS, or LFPs. With this aim, we collected five original research articles focusing on different aspects in developing an explainable BCI, including artifact rejection, feature extraction, classification using explainable algorithms, and hyper-parameter tuning.

Artifact removal is a common topic in the BCI community. In particular, it becomes more challenging to remove artifacts such as electrooculogram (EOG) when the number of recorded EEG signals is limited. To deal-with the over-complete issue while applying independent component analysis (ICA) to remove EOG artifacts from single-channel EEG recordings, Hu et al. proposed a method called DWT-CEEMDAN-ICA, in which a complete empirical mode decomposition method proposed by Torres et al. (2011) that can adapt to noise (CEEMDAN) is used to decompose the discrete wavelet transformation output of the raw EEG signals, ICA is then applied on the decomposed intrinsic mode functions (IMFs) to identify and remove EOG artifacts. This approach might be of interest to those dealing with EOG artifacts in EEG recordings, especially for those focusing on single-channel EEG-based BCIs, further evaluation of the effectiveness of this approach on bigger dataset is still needed though.

To improve the generalizability of motor imagery (MI)-based BCIs, Wang et al. proposed an EEG joint feature classification algorithm based on instance transfer and ensemble learning. In their method, spatial and frequency domain features are extracted through common spatial pattern (CSP, Ramoser et al., 2000) and power spectral density (PSD) analysis, then the MI classification is achieved using an ensemble learning algorithm based on kernel mean matching (KMM, Huang et al., 2007) and adaptive enhancement of transfer learning (TrAdaBoost, Dai et al., 2007). Experimental results using BCI Competition IV Dataset 2a and 2b showed that their method achieved higher decoding accuracies compared with some state-of-the-art methods, demonstrated its effectiveness. However, as the authors acknowledged, this method may not be applicable to other EEG data such as the P300 event-related potential.

Recently, researchers interested in BCI studies have paid great attention to deep leaning-based approaches, in particular, convolutional neural networks (CNN)-based methods. However, there tends to be a trade-off between the accuracy and the interpretability of the trained models, and in many BCI applications, the former is more important. Focusing on this trade-off, Shibu et al. proposed an explainable artificial intelligence (xAI) system that attempts to decompose the CNN model's output onto the input variables (i.e., channels) of fNIRS signals recorded during motor execution or motor imagery. Specially, a method called DeepShap is applied to compute the shapley values, which are further used to explain the model's output (Lundberg and Lee, 2017; Alsuradi et al., 2020). In contrast, Rodriguez et al. took another path and systematically compared the accuracy of decoding movement states and the interpretability between end-to-end CNN-based methods and feature-based methods, i.e., a support vector machine (SVM), using both simulated and real LFP data recorded from ET patients. The synthetic data consisted of 7 different oscillatory patterns including power changes in the beta and gamma bands, beta waveform sharpness, non-linear phase, theta-gamma phase amplitude coupling (PAC), cross-channel phase shifts, and beta burst length. The LFP data were recorded from the bilateral ventra intermediate (VIM) nucleus of the thalamus of ET patients while they perform self-paced upper limb movement tasks (He et al., 2021). The experimental results suggested that end-to-end deep leaning-based methods can yield superior performance, especially in cases where the underlying features of interest are not well-known or not easily captured by standardized feature extraction pipelines. These studies highlighted and made important contributions on the interpretability of CNN-based methods, which may be particularly interesting to those focusing on clinical applications of BCI such as adaptive deep brain stimulation (aDBS).

The performance and interpretability of a BCI system is both somehow determined and represented by a set of hyper-parameters optimized in the trained models. In the last paper collected in this Research Topic, Martineau et al. introduced a Bayesian optimization (BO)-based pipeline for automatic hyper-parameter tuning that is applicable through the entire decoding process, including feature extraction, channel selection, classification, and state-transition stages. The authors compared the proposed method with five other real-time feature extraction methods paired with four classifiers to decode voluntary movement asynchronously based on LFPs recorded with DBS electrodes implanted in the sub-thalamic nucleus (STN) of PD patients, and demonstrated the effectiveness of the proposed pipeline. This study provides a promising solution to deal with the challenges surrounding hyper-parameter tuning while developing BCI systems.

We believe explainable and advanced intelligent processing is an important perspective while developing BCI systems, which can help to improve the performance and interpretability of the developed systems, and will further facilitate the understanding of disease mechanisms (Figure 1). In collating a few good examples of this, we hope this special topic can act as a resource for those interested in this topic, trigger further discussion, and eventually push forward development in this area.

**Figure 1:**
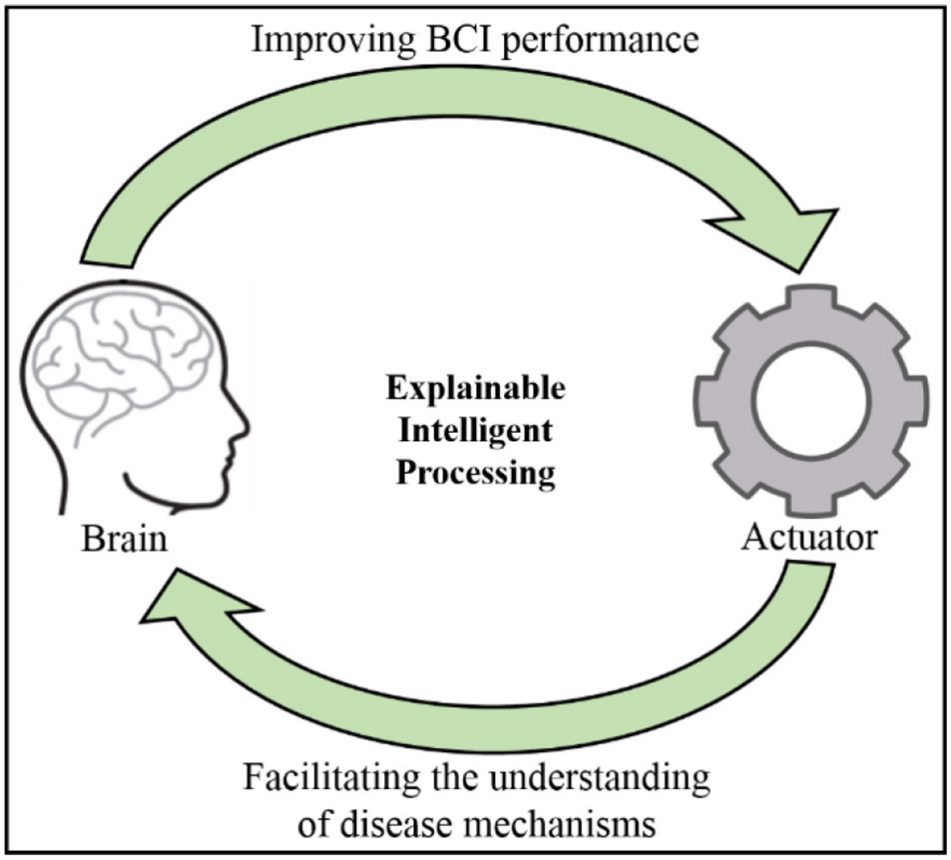
Improving brain-computer interface (BCI) performance and facilitating the understanding of disease mechanisms using explainable intelligent algorithms.

## Author contributions

XX: Conceptualization, Project administration, Writing—review and editing. DZ: Writing—review and editing. TY: Writing—review and editing. YD: Writing—review and editing. ID: Writing—review and editing. SH: Conceptualization, Visualization, Writing—original draft, Writing—review and editing.

## References

[B1] Alsuradi H. Park W. Eid M. (2020). “Explainable classification of EEG data for an active touch task using shapley values,” in HCI International 2020 – Late Breaking Papers: Multimodality and Intelligence 6, eds C. Stephanidis, M. Kurosu, H. Degen and L. Reinerman-Jones (Cham: Springer International Publishing) 406–416. 10.1007/978-3-030-60117-1_30

[B2] Dai W. Qiang Y. Xue G. Yong Y. (2007). “Boosting for transfer learning,” in Proceedings of the 24th International Conference on Machine Learning (Corvalis. OR) 20–24. 10.1145/1273496.1273521

[B3] He S. Baig F. Mostofi A. Pogosyan A. Debarros J. Green A.. (2021). Closed-loop deep brain stimulation for essential tremor based on thalamic local field potentials. Mov. Disord. 36, 863–873. 10.1002/mds.28513 33547859 PMC7610625

[B4] Heldman D. A. Wang W. Chan S. S. Moran D. W. (2006). Local field potential spectral tuning in motor cortex during reaching. IEEE Trans. Neur. Syst. Rehab. Eng. 14, 180–183. 10.1109/TNSRE.2006.875549 16792288

[B5] Huang J. Smola A. Gretton A. Borgwardt K. Schõlkopf B. (2007). “Correcting sample selection bias by unlabeled data,” in Advances in Neural Information Processing Systems. 10.7551/mitpress/7503.003.0080

[B6] Lundberg S. M. Lee S. I. (2017). A unified approach to interpreting model predictions. Adv. Neural Inform. Process. Syst. 30, 4765–4774. 10.48550/arXiv.1705.07874

[B7] Opri E. Cernera S. Molina R. Eisinger R. S. Cagle J. N. Almeida L.. (2020). Chronic embedded cortico-thalamic closed-loop deep brain stimulation for the treatment of essential tremor. Sci. Transl. Med. 12, eaay7680. 10.1126/scitranslmed.aay7680 33268512 PMC8182660

[B8] Ramoser H. Muller-Gerking J. Pfurtscheller G. (2000). Optimal spatial filtering of single trial EEG during imagined hand movement. IEEE Trans. Rehabil. Eng. 8, 441–446. 10.1109/86.895946 11204034

[B9] Torres M. E. Colominas M. A. Schlotthauer G. Flandrin P. (2011). “A complete ensemble empirical mode decomposition with adaptive noise,” in 2011 IEEE International Conference on Acoustics, Speech and Signal Processing (ICASSP) (IEEE) 4144–4147. 10.1109/ICASSP.2011.5947265

[B10] Wolpaw J. R. Birbaumer N. McFarland D. J. Pfurtscheller G. Vaughan T. M. (2002). Brain-computer interfaces for communication and control. Clin. Neurophysiol. 113, 767–791. 10.1016/S1388-2457(02)00057-3 12048038

